# Tattoo free setup for partial breast irradiation: A feasibility study

**DOI:** 10.1002/acm2.12557

**Published:** 2019-04-04

**Authors:** Rachel B. Jimenez, Estelle Batin, Drosoula Giantsoudi, William Hazeltine, Kendell Bertolino, Alice Y. Ho, Shannon M. MacDonald, Alphonse G. Taghian, David P. Gierga

**Affiliations:** ^1^ Department of Radiation Oncology Massachusetts General Hospital Boston MA USA; ^2^ Department of Radiation Oncology UMC Groningen Proton Therapy Centre Groningen Netherlands

**Keywords:** APBI, breast radiotherapy, surface imaging, tattoos

## Abstract

**Purpose:**

Patients undergoing external beam accelerated partial breast irradiation (APBI) receive permanent tattoos to aid with daily setup alignment and verification. With the advent of three‐dimensional (3D) body surface imaging and two‐dimensional (2D) x‐ray imaging‐based matching to surgical clips, tattoos may not be necessary to ensure setup accuracy. We compared the accuracy of conventional tattoo‐based setups to a patient setup without tattoos.

**Materials/methods:**

Twenty consecutive patients receiving APBI at our institution from July 10, 2017 to February 13, 2018 were identified. All patients received tattoos per standard of care. Ten patients underwent setup using tattoos for initial positioning followed by surface imaging and 2D matching of surgical clips. The other ten patients underwent positioning using surface imaging followed by 2D matching without reference to tattoos. Overall setup time and orthogonal x‐ray‐based shifts after surface imaging per fraction were recorded. Shift data were used to calculate systematic and random error.

**Results:**

Among ten patients in the “no tattoo” group, the average setup time per fraction was 6.83 min vs 8.03 min in the tattoo cohort (*P *< 0.01). Mean 3D vector shifts for patients in the “no tattoo” group were 4.6 vs 5.9 mm in the “tattoo” cohort (*P* = NS). Mean systematic errors in the “no tattoo” group were: 1.2 mm (1.5 mm SD) superior/inferior, 0.5 mm (1.6 mm SD) right/left, and 2.3 mm (1.9 mm SD) anterior/posterior directions. Mean systematic errors in the “tattoo” group were: 0.8 mm (2.2 mm SD) superior/inferior, 0.3 mm (2.5 mm SD) right/left, and 1.4 mm (4.4 mm SD) anterior/posterior directions. The random errors in the “no tattoo” group ranged from 0.6 to 0.7 mm vs 1.2 to 1.7 mm in the “tattoo” group.

**Conclusions:**

Using both surface imaging and 2D matching to surgical clips provides excellent accuracy in APBI patient alignment and setup verification with reduced setup time relative to the tattoo cohort. Skin‐based tattoos may no longer be warranted for patients receiving external beam APBI.

## 1 INTRODUCTION

Current standards of care for patients receiving external beam radiation for breast cancer include the administration of permanent skin‐based tattoos on the chest to aid with daily setup alignment and verification. Many patients dislike the idea of tattoos due to concerns for pain from the procedure, impact on body image, religious or cultural beliefs, or the psychological distress conferred by a permanent reminder of their cancer diagnosis.^1^, ^2^, ^3^ There are even rare reports of allergic reactions to tattoo ink administered for radiotherapy.^4^ Additionally, after completion of radiation, tattoos can be challenging to remove either via excision or multiple laser treatment necessitating multiple appointments, additional discomfort, and cost.^5^ Tattoos using ultraviolet ink have been studied to reduce this burden for patients with promising results, but the added cost and setup time for patients requiring specialized ink and lighting for visualization has discouraged widespread utilization.^6^

With the advent of increasingly complex imaging tools including three‐dimensional (3D) surface imaging systems as well as the widespread utilization of radio‐opaque markers that are placed into the surgical bed to delineate the radiation target,^7^, ^8^, ^9^ tattoos may no longer be necessary to ensure setup accuracy. In many institutions, patients with early stage breast cancer have the option to undergo accelerated partial breast irradiation (APBI), in which the radiation target is the surgical bed with margin.^10^, ^11^, ^12^ Daily setup verification in these patients routinely includes a multi‐step process beginning with laser light alignment to tattoos followed by surface imaging and orthogonal x‐rays to align to the patient's surgical clips or other internal radio‐opaque markers. However, to‐date there are no studies demonstrating that tattoos can be avoided in the delivery of breast cancer radiotherapy and nearly all radiation treatment centers continue to utilize tattoos for daily patient setup and positioning. In the following study, we compared the accuracy of this conventional tattoo‐based setup in patients receiving external beam APBI to the identical process without reference to tattoos. Our hypothesis was that patient positioning would be equally accurate and that the patient's overall treatment time would be more efficient than a tattoo‐based setup.

## 2 METHODS AND MATERIALS

**Figure 1:**
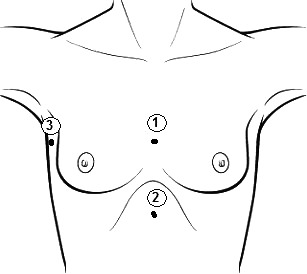
Institutional tattoo setup per current standards of care.

Twenty consecutive patients receiving APBI for early stage breast cancer at Massachusetts General Hospital from July 10, 2017 to February 13, 2018 were identified. All patients initially underwent breast conserving surgery and had surgical clips placed in the wall of the lumpectomy cavity at the time of surgery to demarcate the tumor bed. All patients met ASTRO consensus guidelines for receipt of APBI. Subsequently, patient underwent a free breathing computed tomography (CT) simulation for radiotherapy and received conventional tattoos per standard of care. At our institution, breast patients receive three tattoos: tattoo 1 is located at chest midline at the approximate level of the nipple, tattoo 2 is located at chest midline approximately ten centimeters inferior to tattoo 1, and tattoo 3 is located at the mid‐axillary line on the ipsilateral chest wall, level to tattoo 1 (Fig. 1). After treatment planning per standard of care, all patients received either daily or twice daily radiotherapy to the surgical bed plus a 1.5–2 cm margin for a total of 9–10 total fractions, per institutional dose and fractionation schemes for APBI.^13^

Of the twenty patients identified, the initial ten were deemed the “tattoo” group and each underwent daily setup verification with reference to their tattoos per standard protocol. This consisted of laser light alignment to the skin‐based tattoos for initial positioning followed by further verification with 3D surface imaging and finally, orthogonal kV x‐rays with two‐dimensional (2D) matching of the surgical clips to the reference digitally reconstructed radiograph (DRR). The second group of ten patients was deemed the “no tattoo” group and they underwent daily setup verification without reference to their tattoos. The “no tattoo” group underwent initial positioning using 3D surface imaging alone followed by orthogonal x‐rays with 2D matching to the surgical clips using the DRR. In neither group were MV port films used for patient setup, but MV images were obtained on the first day of treatment for both groups once alignment was complete to confirm the proper aperture shape.

Each patient and their intended setup method was identified in advance of treatment planning and the participating radiation therapists were provided specific instructions for each patient in advance of treatment to ensure adherence to the intended setup schema. Setup time for each delivered fraction was measured from the time the patient entered the treatment room to the beginning of x‐ray imaging.

**Figure 2:**
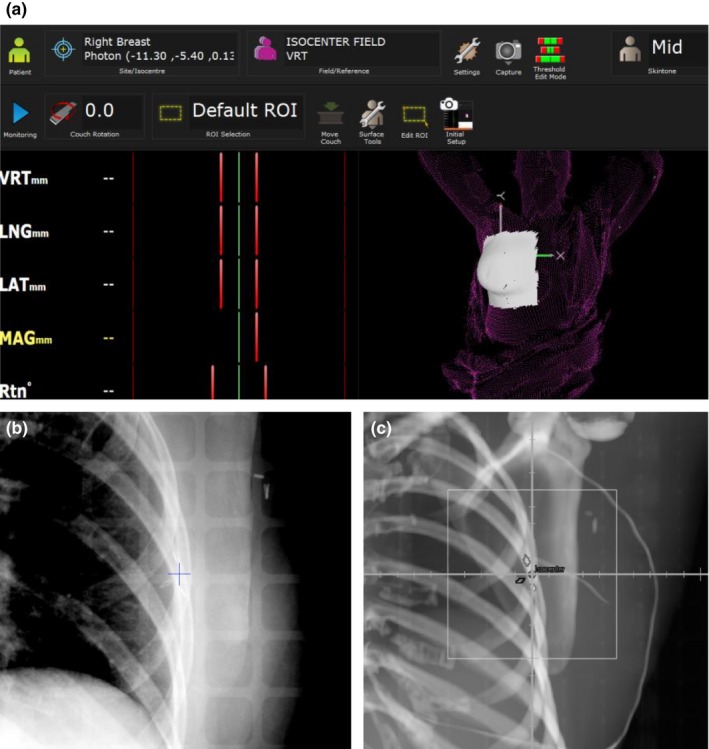
(a) Region of interest (ROI) using AlignRT for APBI with (b) associated example daily orthogonal x ray, and (c) digitally reconstructed radiograph.

Surface imaging was performed using the AlignRT system (VisionRT, London, UK). For patients with tattoos, the reference surface was captured at the first fraction with the AlignRT system once the patient was in the treatment position (confirmed by x‐ray imaging) and this reference surface was then used for all subsequent treatments. For patients without tattoos, the reference surface for the first fraction was based on a surface constructed from the skin contour from the planning CT and included the planned isocenter location. A new reference surface was captured with the AlignRT system once the patient was in the treatment position and this reference image was then used for all subsequent treatments. A region of interest (ROI) was defined which included the breast and an appropriate amount of surrounding tissue based on the site‐specific system's guidelines [Fig. 2(a)]. For the “no tattoo” group, the patient was positioned on the LINAC couch and clinically straightened with the initial isocenter location placed approximately in the center of the breast. During this step, the room lasers were turned off. The light field in the head of the gantry was turned on when the patient was placed on the table, but the jaws were in open position so that the treatment field was not projected onto the patient nor was the light field graticule used as reference for patient alignment. Surface imaging was then performed using continuous monitoring to align the patient to the planned isocenter location. The therapists were instructed to minimize the real‐time deltas (the mismatch between the current and reference images) but residual setup errors up to a 3 mm tolerance were allowed. Correction for patient rotation was not performed using couch rotations (YAW, ROLL, and PITCH) but the patient was manually rotated if large rotations (typically exceeding 3°) were indicated by the surface imaging system. The real‐time deltas were recorded throughout the alignment process. Variations in initial patient positioning were assessed by examining the initial shift (acquired after positioning either with or without tattoos) extracted from the real‐time surface monitoring data.

The surgical clips were outlined in the planning CT and denoted on the reference DRR. X‐ray image registration was performed by aligning the clip position in the daily orthogonal kV x rays to their position in the reference DRR [Figs. 2(b) and 2(c)]. Any residual setup corrections from x‐ray imaging after 3D surface imaging were recorded for each fraction. Setup corrections consisted of translations only; no couch rotations were performed. If any shift indicated by x ray was greater than 5 mm, the patient was re‐imaged with x rays to confirm the treatment position after applying the shifts. The number of fractions with shifts greater than or equal to 5 mm was also tracked for each patient group. The x‐ray shift data were corrected by subtracting any residual alignment error from surface imaging. The x‐ray shift data were subsequently used to calculate the 3D vector setup error.

Setup error for both initial positioning (either with or without tattoos) and for x‐ray imaging (residual errors after surface imaging) were quantified using random and systematic errors. In a patient cohort, for a given translation direction, S_p_ is defined as the mean setup error for each patient, and the mean of all S_p_ values, over all patients, is the group mean, *μ*. The standard deviation of *μ* is Σ, and both *μ* and Σ quantify systematic errors. In addition, if *σ*_p_ is the standard deviation of the translation variations for each patient, then average of all *σ*_p_, or *σ*, quantifies the random errors.^14^, ^15^ Differences in setup time, mean (*μ*) systematic errors, and the 3D vector errors between the “tattoo” and “no‐tattoo group” were tested for statistical significance as were differences between the random and systematic standard deviations (*σ* and Σ) using the Mann–Whitney or Brown–Forsythe tests, as appropriate.

## 3 RESULTS

The mean (SD) setup time for the “no tattoo” cohort was 6.83 min (0.75 min SD) vs 8.03 min (0.74 min SD) for the “tattoo” cohort. The difference in setup time for the two groups was statistically significant (*P* < 0.01).

**Figure 3:**
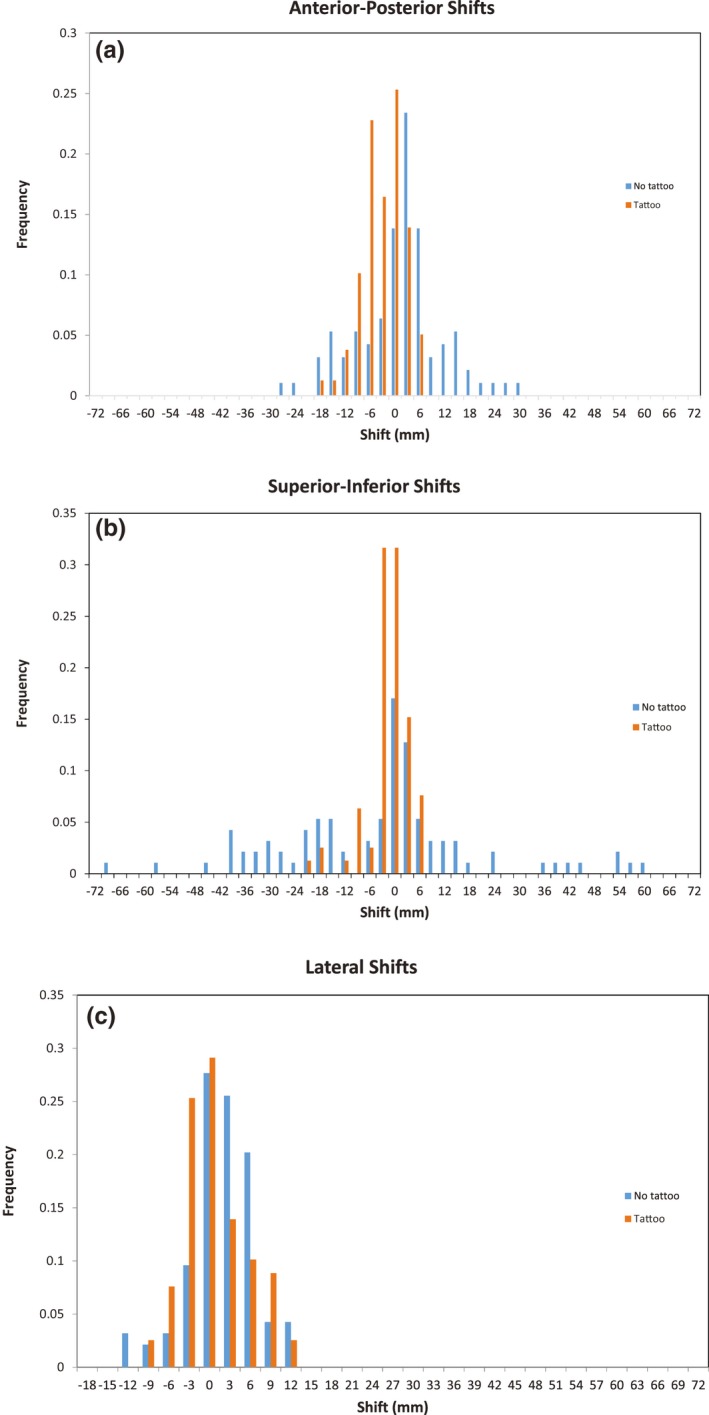
Initial setup errors detected by surface imaging.

Shifts for initial setup were available for 79 and 94 fractions for the groups with and without tattoos, respectively. Histogram data for the initial setup errors detected by a surface imaging, either with or without the tattoo‐based setup, are shown in Fig. 3. The setup errors for initial positioning are tabulated in terms of random and systematic errors in Table 1. Mean 3D vector shifts for patients in the “no tattoo” group were statistically different from the patients in the “tattoo” group, but the individual vector directions were not different between the two groups. The random and systematic errors were also larger in the no tattoo group and in some cases were statistically significant with the largest errors identified in the superior–inferior direction. This appears consistent with the expectation that initial positioning would be less accurate and precise given the absence of tattoos to guide patient alignment.

**Table 1:** Random and systematic errors for initial patient positioning, either with or without tattoos

Data in mm	Tattoo group				No‐tattoo group			
	Sup/inf	Ant/post	Left/right	3D	Sup/inf	Ant/post	Left/right	3D
Mean (*μ*)	−3.0	−4.3	−1.0	8.9^a^	−5.0	0.1	0.4	20.0^a^
Std dev (Σ)	4.2^a^	3.5	3.8	3.4^a^	20.8^a^	9.2	3.3	14.1^a^
Std dev (*σ*)	1.5^a^	1.8	1.2	1.9^a^	5.8^a^	2.9	1.1	5.5^a^

a Indicates statistically significant difference between the two patient groups.

**Figure 4:**
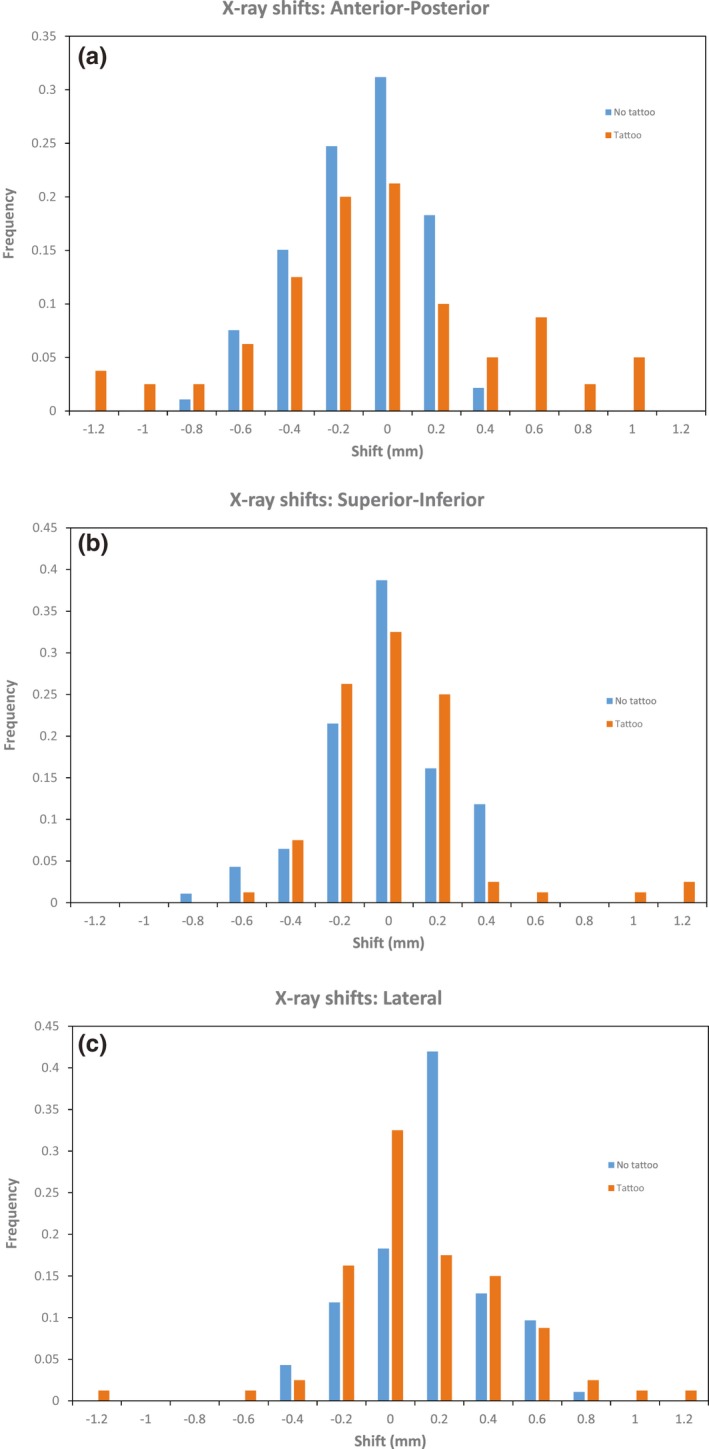
Setup errors detected by x rays following surface imaging.

For shifts detected after x‐ray imaging, data were available for 80 and 93 fractions for the groups with and without tattoos, respectively. Both patient groups had a similar percentage of fractions with raw x‐ray shifts in any one direction greater than 5 mm, (36% and 38% for the “tattoo” and “no tattoo” group, respectively). Mean 3D vector shifts for patients in the “no tattoo” group were 4.6 mm vs 5.9 mm in the “tattoo” cohort. This difference was not statistically significant. Random and systematic errors for both patient groups are summarized in Table 2/Fig. 4. There were no statistically significant differences in the random and systematic errors.

**Table 2:** Random and systematic errors determined by x‐ray imaging after surface imaging setup, corrected for any residual setup errors from surface imaging

Data in mm	Tattoo Group				No‐tattoo Group			
	Sup/inf	Ant/post	Left/right	3D	Sup/inf	Ant/post	Left/right	3D
Mean (*μ*)	0.8	−1.4	0.3	5.9	1.2	2.3	0.5	4.6
Std dev (Σ)	2.2	4.4	2.5	2.5	1.5	1.9	1.6	1.5
Std dev (*σ*)	1.2	1.5	1.6	1.7	0.7	0.7	0.6	0.6

## 4 DISCUSSION

Results from this study suggest that among a cohort of APBI patients receiving external beam radiation, patient positioning appears equally accurate when tattoos are not utilized for daily setup verification if a combination of 3D surface imaging and 2D matching of internal surgical clips is performed. The hypothesis is supported by the minimal observed differences in the setup errors (measured by x‐ray clip alignment) between the two groups. These results are likely because surface imaging provides fairly accurate setup, regardless of whether tattoos are used. Such data are consistent with previous publications that indicate that the differences between surface imaging and clips for APBI are small, but that there are demonstrated improvements in APBI setup with the use of surface imaging compared to a laser‐based tattoo setup alone.^7^, ^15^, ^16^

As expected, the initial setup errors for patients, as quantified by initial surface imaging results, are, overall, larger for the “no tattoo” patient group. This is as expected as these patients are simply clinically straightened and aligned to an approximated isocenter location without the benefit of tattoos. These initial setup errors are subsequently corrected by surface imaging prior to x‐ray imaging and do not appear to affect the final accuracy of the patient position based on clips.

Furthermore, the data indicates that patients’ overall time in the treatment room was abbreviated when tattoos were not referenced. For the “no tattoo” group the initial setup times were likely shortened due to some redundancy in patient alignment when using lasers and skin marks and when using surface imaging. While the overall treatment time length was modestly shorter, less time in the treatment room may enhance the patient's experience with treatment and, when considered across multiple patients, could permit greater utilization of the treatment machine in a busy clinical practice.

It should be noted that for this study, patients were given standard skin tattoos although the therapists were instructed not to use them during setup. It is possible, although unlikely, that results would differ if the patients did not have tattoos and this will be tested in a future study. Nevertheless, this suggests that the placement of skin‐based tattoos may no longer be necessary in APBI patients and that patients can receive equally accurate treatments without the inconvenience and permanent disfigurement created by tattoos. Although subtle, such a change in practice represents an evolution in radiotherapy treatment delivery as tattoos have been a mainstay of treatment for decades. Yet, tattoos continue to be a psychological burden to many breast cancer patients within our own clinical practice as well as reported via online support groups for breast cancer highlighting patient's dislike of, and distress over, receiving permanent tattoos.^1^, ^2^, ^3^, ^17^ Now, with evolving technology, we have the opportunity to improve patient quality‐of‐life without sacrificing the treatment fidelity. This is particularly relevant for APBI patients who often elect to pursue APBI to minimize the inconvenience of a lengthier treatment and who, as a result, may be more sensitive to the idea of permanent tattoos than other patients.

Future work will focus on implementing a tattoo‐free setup among our APBI patients to evaluate setup fidelity. We also plan to expand this concept of a tattoo‐free setup to the larger population of patients receiving whole breast radiotherapy with tangents.

## 5 CONCLUSION

Among a cohort of early stage breast cancer patients receiving APBI, using a combination of surface imaging and 2D matching to surgical clips provided excellent accuracy in patient alignment and setup verification compared to a tattoo‐based setup, with reduced setup time. Skin‐based tattoos may no longer be warranted for patients receiving external beam APBI. Future investigation of a tattoo‐free setup for patients receiving whole breast radiation is forthcoming.

## CONFLICT OF INTEREST

Dr. Gierga has previously received speaking honoraria for VisionRT. The remaining authors have no conflict of interest to disclose.
